# Neuroprotective effect of microglia against impairments of auditory steady-state response induced by anti-P IgG from SLE patients in naïve mice

**DOI:** 10.1186/s12974-020-1716-9

**Published:** 2020-01-23

**Authors:** Xuejiao Wang, Yingzhuo Li, Zijie Li, Jinhong Li, Jingyi Xu, Pingting Yang, Ling Qin

**Affiliations:** 1 Department of Physiology, China Medical University, Shenyang, 110001 People’s Republic of China; 2 Department of Rheumatology and Immunology, First Affiliated Hospital, China Medical University, Shenyang, 110001 People’s Republic of China

**Keywords:** Electroencephalogram, Evoked potentials, Autoantibody, Encephalopathy, Microglia, Phagocytosis

## Abstract

**Objective:**

Autoantibodies against ribosomal P proteins (anti-P antibodies) are strongly associated with the neuropsychiatric manifestations of systemic lupus erythematosus (NPSLE). The present study was designed to assess whether anti-P antibodies can induce abnormal brain electrical activities in mice and investigate the potential cytopathological mechanism.

**Methods:**

Affinity-purified human anti-ribosomal P antibodies were injected intravenously into mice after blood–brain barrier (BBB) disruption. The auditory steady-state response (ASSR) was evaluated based on electroencephalography (EEG) signals in response to 40-Hz click-train stimuli, which were recorded from electrodes implanted in the skull of mice. Immunofluorescence staining was used to examine the morphology and density of neurons and glia in the hippocampus and cortex. The presence of apoptosis in the brain tissues was studied using the TUNEL assay. A PLX3397 diet was used to selectively eliminate microglia from the brains of mice.

**Results:**

Circulating anti-P antibodies caused an enhancement of the ASSR and the activation of microglia through the disrupted BBB, while no obvious neural apoptosis was observed. In contrast, when microglia were depleted, anti-P antibodies induced a serious reduction in the ASSR and neural apoptosis.

**Conclusion:**

Our study indicates that anti-P antibodies can directly induce the dysfunction of auditory-evoked potentials in the brain and that microglia are involved in the protection of neural activity after the invasion of anti-P antibodies, which could have important implications for NPSLE.

## Introduction

Diffuse brain dysfunction without overt brain inflammation frequently occurs in systemic lupus erythematosus (SLE) and might involve pathogenic autoantibodies, especially those against neuronal surface components [1, 2]. Previous studies have shown that anti-ribosomal P (anti-P) antibodies are associated with neuropsychiatric SLE (NPSLE) [3, 4]. Anti-P antibodies are specific markers for SLE [5] and are detected predominantly in patients during the active phases of SLE [6, 7]. An association between circulating anti-P antibodies in the blood and NPSLE manifestations has been confirmed [8–12]. Anti-P antibodies have also been found in the cerebrospinal fluid of patients with NPSLE, indicating blood–brain barrier (BBB) permeation [11, 13].

Several animal experiments have been conducted to reveal the pathogenetic roles of anti-P antibodies in the central nervous system (CNS). The results show that the passive transfer of human anti-P antibodies to mice can cause cognitive, emotional, and memory dysfunction [14, 15]. The extent of anti-P antibody-induced dysfunction is dependent on the concentration of these antibodies. This may be attributable to the fact that anti-P antibodies bind to neuronal surface proteins [16], leading to calcium influx and neural apoptosis or functional perturbation [15, 16].

However, there is still a lack of direct in vivo evidence of the pathogenic effect of anti-P antibodies on neural electrophysiological activity. Although Gaburo et al. recently reported that the intraventricular injection of human anti-P antibodies in rats can induce electroencephalogram (EEG) alterations [17], the EEG results were not quantitatively analyzed and cannot be used as a marker to reflect psychiatric changes.

Clinically, one commonly used method of evaluating brain electrical activity is the auditory steady-state response (ASSR), which is an EEG signal entrained to periodic auditory stimuli (a train of clicks) [18]. The power (magnitude) of the ASSR can reflect the functional integrity of the neural circuits that support synchronization across frequencies [19, 20]. For this, the ASSR can be used to evaluate the sensory and cognitive functions of the CNS [21–24]. EEG measurement of the ASSR, particularly in the gamma frequency range (30–80 Hz), has been commonly used in the clinical examination of mental illness [25–28] and in neuropharmacological experiments in animal models [29–31]. To further investigate how anti-P antibodies disturb neural functions, we recorded the ASSR from mice using chronic electrodes implanted in the skull over the primary auditory cortex (A1). A1 is the first station in cortical auditory processing and plays a key role in sound representation and auditory perception [32, 33]. We recorded the ASSRs from A1 of each mouse to monitor changes in the EEG signal induced by the passive transfer of human anti-P antibodies to the mice. By combining electrophysiological and histochemical methods, we revealed the detailed effects of anti-P antibodies on brain electrical activity and the protective role of microglia.

## Materials and methods

### Mice

Experiments were performed using 8–12-week-old C57BL/6 male mice (Vital River Laboratory, Beijing, China). All animals were maintained in standard animal cages under conventional laboratory conditions (12-h/12-h light/dark cycle, 22 °C) with ad libitum access to food and water. The animals were maintained and treated in compliance with the policies and procedures detailed in the “Guide for the Care and Use of Laboratory Animals” of the National Institutes of Health. The animal experimental protocols of the “Guide” and the treatment procedures were reviewed and approved by the Animal Care and Use Committee of China Medical University (No. KT2018060). All surgeries were performed under anesthesia, and all efforts were made to minimize animal suffering.

### Source of human sera

Serum samples from six patients who fulfilled the American College of Rheumatology criteria for SLE [34] and five healthy individuals were used in the present study. Patients who showed severe psychiatric disturbances and high levels of anti-P antibodies (92 ± 25 IU/ml, *n* = 6) were selected from the 150 SLE patients attending the Department of Rheumatology and Immunology in the First Affiliated Hospital of China Medical University. The presence of anti-P antibodies was further tested by western blot analysis using the SDEDMGFGLFD peptide of the 11 carboxy-terminal residues of ribosomal P proteins as the antigen [16]. The serum level of anti-P antibodies in healthy individuals was 12 ± 10 IU/ml (*n* = 5).

### Purification of anti-P antibody IgG

IgG was isolated from the pooled sera of patient or healthy subjects using protein A-resin (genScript, Piscataway, NY) and concentrated using Amicon Ultra Centrifugal Filter Units (Millipore, Billerica, MA). Anti-P antibody IgG (anti-P IgG) was purified from patients’ IgG using a sepharose column to which the ribosomal P antigen had been conjugated. IgG from healthy individuals was used as a control. The IgG concentration was adjusted to 1.7 mg/ml with buffer for the experiments.

### Electrode implantation

Mice were handled according to the criteria of the ethics committee at our institution. Following a period of 2 weeks of handling at least once a day for 5 min, animals underwent surgery for the long-term implantation of single-wire electrodes. Mice were anesthetized with isoflurane in conjunction with air (3% for induction and 1–2% for maintenance). Atropine sulfate (0.1 mg/kg) was administered at the beginning of the surgery to reduce the viscosity of bronchial secretions. Body temperature was monitored rectally and maintained at 37 °C using a feedback-controlled blanket. After placing the animal in a stereotaxic frame (#68001, RWD Life Science, Shenzhen, China), the skull was exposed. Two stainless screws were separately inserted into A1 of both hemispheres (AP = − 2.3–3.5 mm and ML = + 3.5–4.5 mm) according to a standard mouse stereotaxic atlas. One end of a silver microwire (#785500, A-M Systems, Hofheim, USA) was used as an electrode and fixed to the bone by the screws. The other end of the microwire was soldered to a pin connector, which was secured to the skull using dental acrylic resin. A stainless-steel screw electrode placed over the cerebellum served as a ground. Four additional skull screws were implanted and served as anchors. Animals were allowed to recover for 2 weeks.

### Electrophysiological recordings and sound stimuli

After recovery from surgery, animals were acclimated to a sound-attenuated recording room. Briefly, the animals were transported in their home cages to the recording room, where they were left alone for 5 min. They were then put in a mesh box (40 × 40 × 60 cm) and tethered to the recording system via a flexible cable headstage for 15 min. This procedure was repeated for 4 days. Recording experiments were conducted on the 5th day. The sound stimulus used to assess the ASSR in our experiments was a train of click sounds. The waveform of each click was a rectangular pulse with a 0.2-ms duration, which was repeated at a rate of 40 cycles/s and continued for 0.5 s. The waveforms were generated digitally at a 100-kHz sampling rate using a custom built MATLAB (MathWorks, Natick, MA, USA) program, transferred to an analog signal by a D/A board (PCI-6052E, National Instruments, Austin, Texas, USA), and then played through a loudspeaker (K701, AKG, Vienna, Austria) on top of the recording box. The intensity of the sound stimulus was adjusted to 70 dB SPL when measured from the center of the recording box (Brüel & Kjær Type 2238 Sound Level Meter, Naerum, Danish). In one session, 120 click train trials were presented at random intervals between 4 and 8 s.

### Breakdown of the BBB and the passive transfer of IgG to mice

After completing one session of EEG recording under normal conditions, the mice received IgG by passive transfer. Before IgG transfer, the blood–brain barrier was disrupted using a previously described method [35]. In brief, 50 μg complete Freund’s adjuvant (CFA, Sigma-Aldrich, USA) containing heat-killed H37Ra *Mycobacterium tuberculosis* (Difco, USA) in 50 μl was subcutaneously injected into each of four sites on the hind flank. In addition, mice received an intraperitoneal (i.p.) injection of 200 ng pertussis toxin (PTx, List Biological Laboratories, USA) in 0.2 ml PBS. The injection of PTx was repeated again 3 days later. On the 7th day after the injection of CFA, animals were injected intravenously with PBS (as vehicle) or test or control IgG (200 μg in 0.2 ml PBS). Electrophysiological recording was conducted again 1, 24, 48, and 72 h after the injection of IgG.

### Electrophysiological data acquisition and analysis

EEG signals were acquired using a flexible, low-noise cable connected to the pin connector implanted in the skull of the mice. The microwire output was delivered to a multichannel preamplifier (PBX Preamplifier; Plexon, Dallas, Texas, USA) and then to a digital multichannel acquisition processor (MAP; Plexon). The EEG waveforms were amplified and low-pass filtered using a 300-Hz cutoff frequency and then imported into MATLAB for analysis.

First, the EEG was visually checked to exclude artifacts. The EEG fragments within a 500-ms epoch before the onset of the sound stimulus and 500 ms after stimulus offset were averaged for all trials without artifacts. The mean amplitude from the 500 ms before stimulus onset was used as the baseline value (baseline correction). The EEG spectrum analysis was conducted with a wavelet-based analysis algorithm implemented in custom-written code using the EEGLAB toolbox (https://sccn.ucsd.edu/eeglab/index.php). The power of the evoked EEG spectrum was presented as a relative estimation of the ratio between the values after stimulus onset and the prestimulus values (stimulus/prestimulus).

### Microglial depletion

For the microglial depletion experiments [36, 37], mice were fed a PLX3397 (MedChemExpress) diet (290 ppm; 290 mg/kg chow) for 3 weeks.

### Immunofluorescence

We used another set of mice to examine the effects of anti-P IgG on the morphology of neurons and glial cells. Twenty-four hours after the injection of IgG, mice were anesthetized and perfused transcardially with 10–20 ml of 0.09% NaCl to eliminate the blood and then with 4% paraformaldehyde in 0.1 M phosphate buffer, pH 7.4, to fix the brain. The fixed brain was isolated and postfixed for 2 h in 4% PFA. Twenty-five-micrometer-thick sections were immunostained at room temperature for 1 h with antibodies against mouse neuronal nuclear antigen (NeuN, 1:500, Abcam), glial fibrillary acidic protein (GFAP, 1:500, Abcam), and ionized calcium-binding adaptor molecule-1 (Iba1, 1:100, Abcam) followed by the appropriate fluorescent secondary antibody (1:300, Proteintech). Tissue sections were examined with a fluorescence microscope (BX53, Olympus). NeuN-, GFAP-, and Iba-1-immunonegative areas were defined by hand and quantified using ImageJ. TUNEL staining was performed using a TUNEL Bright Green Apoptosis Detection kit (Vazyme, China). We selected five sections containing the hippocampus between 1.8 and 2.3 mm posterior to bregma at 100-μm intervals from each mouse brain. We selected this brain area because previous studies have demonstrated that intravenously injected anti-P antibodies can enter the brain through the disrupted BBB and react with the hippocampus and surrounding cortices [14–16]. The density of immunopositive cells was determined in the hippocampus and parietal cortex above the hippocampus using ImageJ software. The data from the five sections were averaged to obtain a single data point for each individual mouse. All quantifications were performed in a blinded manner. The data are presented as the area (mm^2^) of the immunonegative area.

### Statistical analysis

Statistical analysis was performed using SPSS for Windows (Chicago: SPSS, Inc.). The data are presented as the mean ± SE. Differences between the results of two groups were detected by Student’s *t* test. Differences between the results of multiple groups were detected using one-way analysis of variance (ANOVA). Each ANOVA that revealed significant effects was followed by Tukey’s post hoc multiple comparisons test. Statistical significance was defined as *p* < 0.05.

## Results

### Circulating anti-P antibodies strengthen the ASSR in mice when the BBB is disrupted

**Fig. 1:**
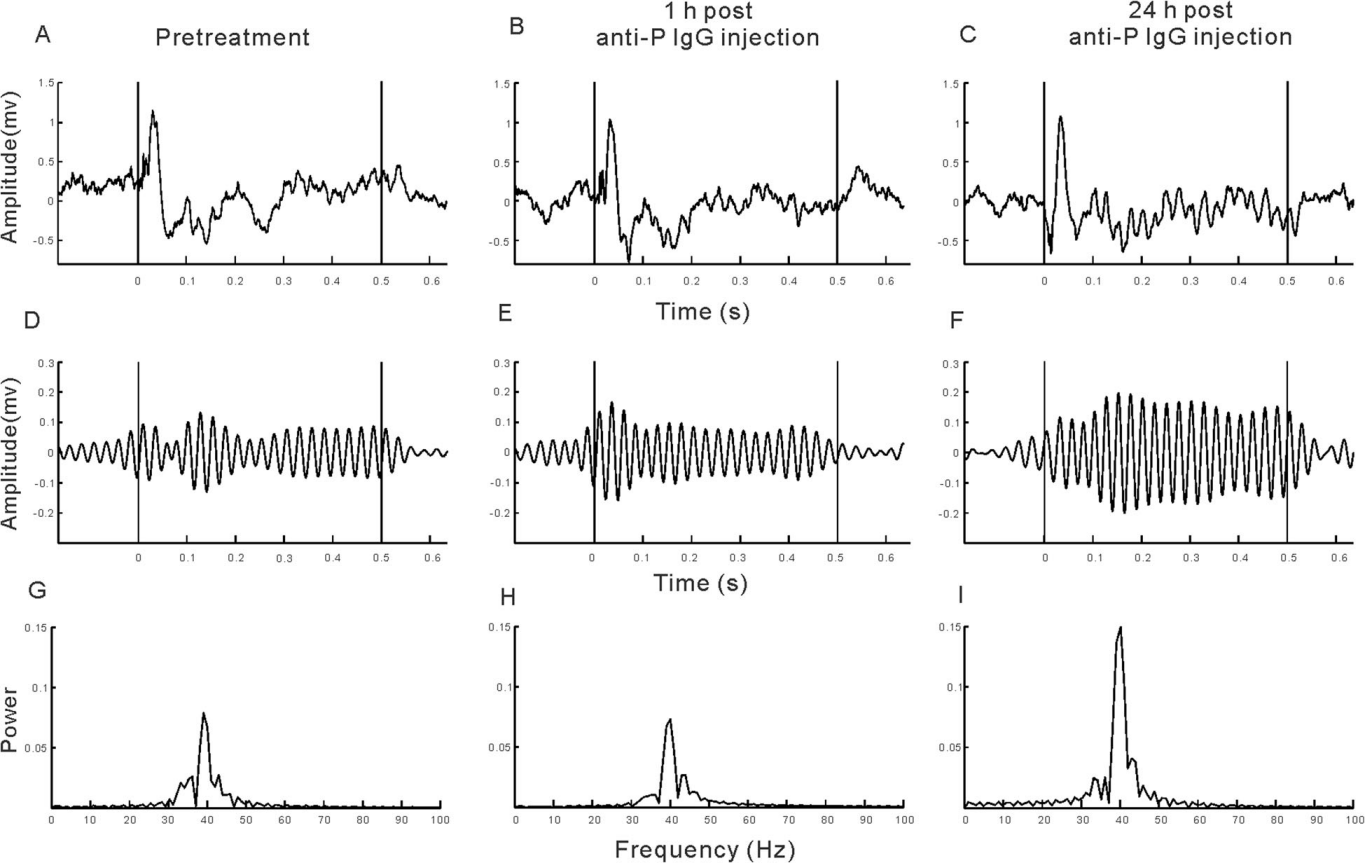
Example of ASSR recorded from one mouse. **a**–**c** Unfiltered ASSR averaged from 120 trials of EEG signals evoked by 40-Hz click stimuli lasting from 0 to 0.5 s. **d**–**f** The same EEG responses filtered with a bandpass filter. **g**–**i** Power spectrum of the filtered EEG responses. Vertical lines mark the onset and offset of sound stimulus

**Fig. 2:**
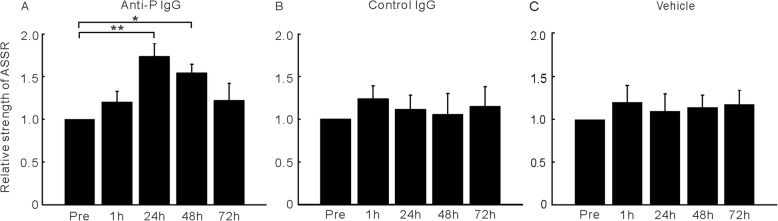
ASSR strength of the anti-P (**a**), control IgG (**b**), and vehicle group (**c**) at different time points. Bars represent the mean of ASSR strength (*n* = 8) relative to the value of pretreatment. Error bar represents SE. ** indicates *p* < 0.01; * indicates *p* < 0.05 (ANOVA and Tukey’s post hoc test)

To explore the neuropathogenic potential of circulating anti-P IgG, we injected anti-P IgG intravenously into a mouse model after the BBB was disrupted [35]. EEG signals from each mouse were recorded before and after IgG treatment. Representative results from one mouse are presented in Fig. 1. Under normal conditions, the EEG showed a large deflection at the onset of the stimulus followed by a stable oscillation synchronized to the 40-Hz click train (Fig. 1a). To compare the EEG signals with the frequency of the stimuli, the EEG signals were filtered with a bandpass filter of 35–45 Hz. The filtered EEG showed a clear oscillation synchronized to the stimulus frequency (Fig. 1d). Power spectrum analyses of the EEG signal also showed a clear peak at 40 Hz, reflecting the strength of the 40-Hz ASSR (Fig. 1g). One hour after anti-P IgG injection, the ASSR recorded from each mouse remained unchanged (Fig. 1b, e, and h), indicating that BBB disruption had no significant effect on ASSR. However, the ASSR was obviously enhanced 24 h after anti-P IgG injection (Fig. 1c, f, and i). Figure 2 shows the mean ASSR strength in the groups of mice that received anti-P IgG, control IgG, or vehicle (*n* = 8 for each group) injection at different time points. Compared to pretreatment, one injection of anti-P IgG significantly increased the ASSR at 24 h. This increase was maintained 48 h after injection and recovered at 72 h (ANOVA and Tukey’s post hoc test, Fig. 2a). In contrast, control IgG or vehicle injection did not significantly change the ASSR (Fig. 2b and c).

### Microglia are activated by anti-P IgG

**Fig. 3:**
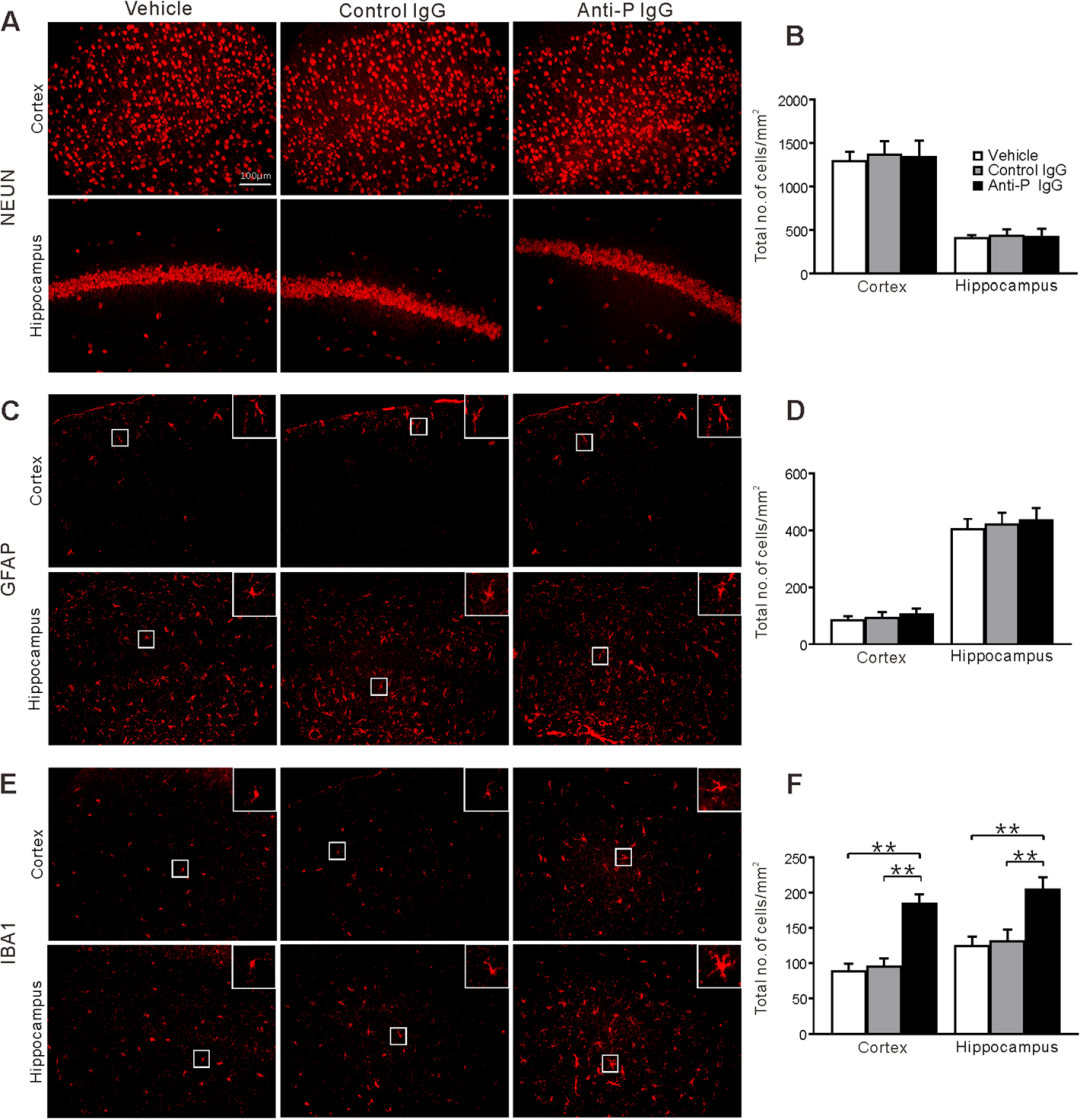
Immunohistochemical analysis of brain section in the cortex and hippocampus following administration of vehicle, control or anti-P IgG (*n* = 6). **a** Representative microphotographs showing NeuN-immunopositive cells in the cortex and hippocampus. **b** Quantitative analysis of the number of NeuN-immunopositive cells in the cortex and hippocampus. Error bar represents SE. There is no significant difference between the groups. **c** Representative microphotographs showing GFAP-immunopositive cells. White rectangle shows an enlarged cell to compare the morphology. **d** Quantitative analysis of the number of GFAP-immunopositive cells. **e** Representative microphotographs showing Iba-1-immunopositive cells. **f** Quantitative analysis of the number of Iba-1-immunopositive cells. ** indicates *p* < 0.01 (ANOVA and Tukey’s post hoc test)

**Fig. 4:**
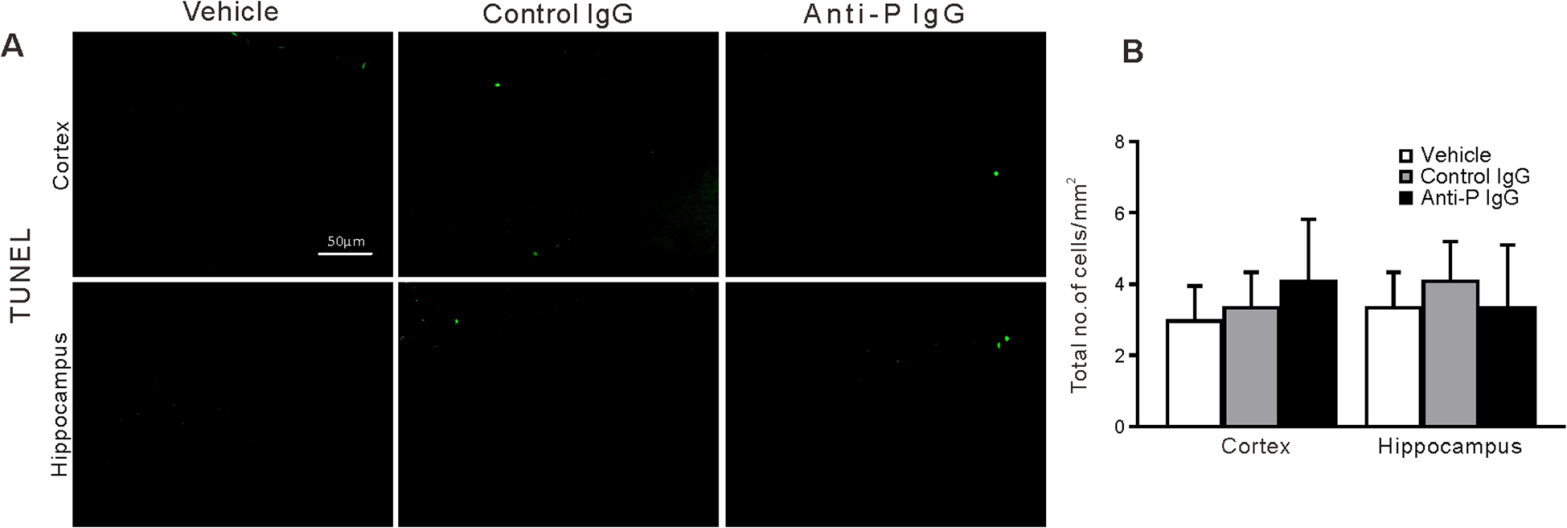
TUNEL staining of brain section in the mouse treated by vehicle, control, or anti-P IgG. **a** Representative section of cortex and hippocampus. **b** Quantitation of the number of TUNEL positive cells. The same format as Fig. 3

We conducted immunofluorescence examination of another set of mice to explore the histological changes induced by anti-P IgG. Twenty-four hours after vehicle, anti-P or control IgG injection, the mice were sacrificed to conduct immunofluorescence analysis on brain slices. We gauged and compared the distribution of cells immunoreactive for markers of neurons (NeuN), astrocytes (GFAP), and microglia (Iba-1) in the cortex and hippocampus of mice in different groups (*n* = 6 for each group). Figure 3 shows immunofluorescence for NeuN, GFAP, and Iba1. The density and morphology of NeuN-immunopositive neurons and GFAP-immunopositive astrocytes were similar between the vehicle, control IgG, and anti-P IgG injection conditions. However, the density of Iba-1-immunopositive microglia was higher in the mice that received anti-P IgG injection than in the mice that received vehicle or control IgG injection. Furthermore, anti-P IgG injection induced microglia to show an activated morphology (reduced number of ramifications and thickened processes). The TUNEL assay did not reveal significant signs of apoptosis in the three groups (Fig. 4).

### Anti-P IgG results in impairments in the ASSR after the depletion of microglia

**Fig. 5:**
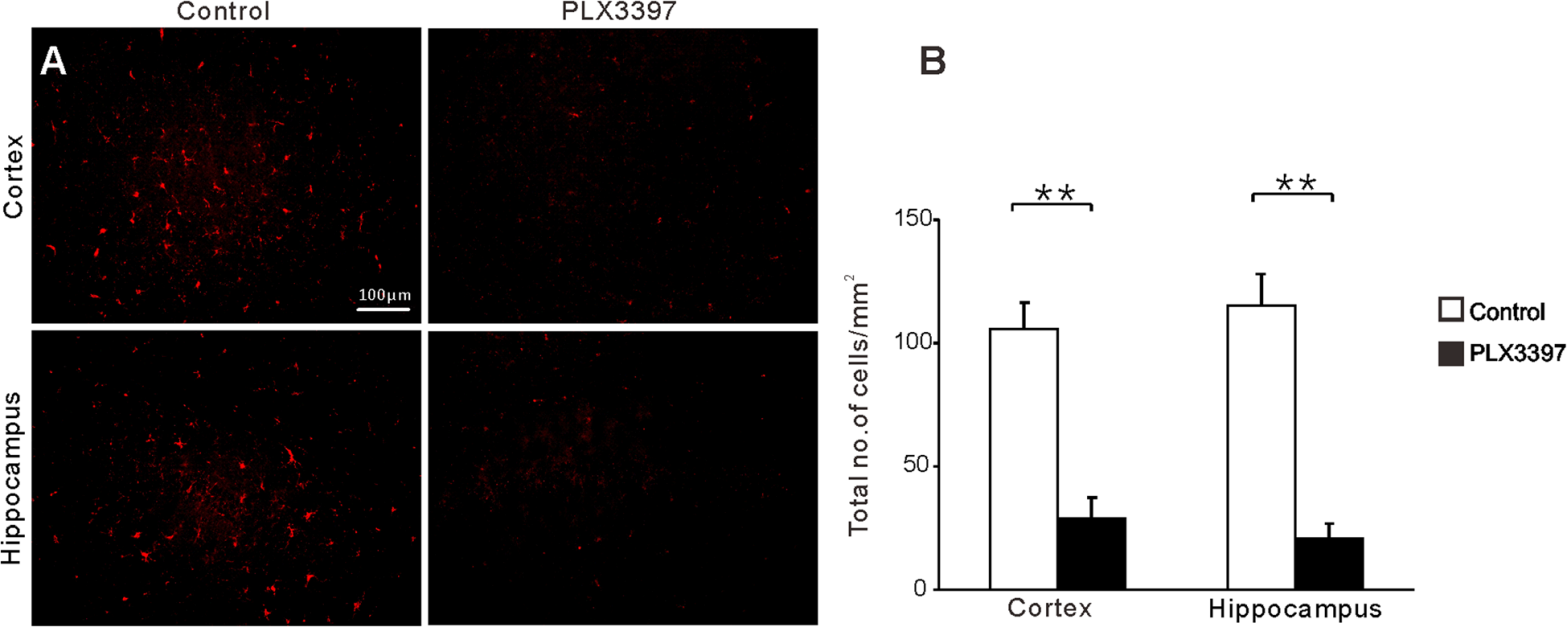
CSF1R inhibition eliminates microglia from the mouse brain. **a** Representative Iba-1 immunofluorescent staining from the cortex and hippocampus in control and PLX3397-treated mice. **b** Quantification of microglia in mice after control or PLX3397 diet. ** indicates *p* < 0.01 (Student’s *t* test, *n* = 6)

To elucidate the role of activated microglia in response to the transfer of anti-P IgG, we used a colony-stimulating factor 1 receptor (CSF1R) kinase inhibitor to deplete microglia in mice [36]. The administration of a chow diet containing the CSF1R antagonist PLX3397 (290 mg/kg chow) for 3 weeks resulted in an almost complete elimination of microglia from the brains of the mice, as confirmed by the absence of red fluorescent protein (Iba1)-positive cells (Fig. 5).

**Fig. 6:**
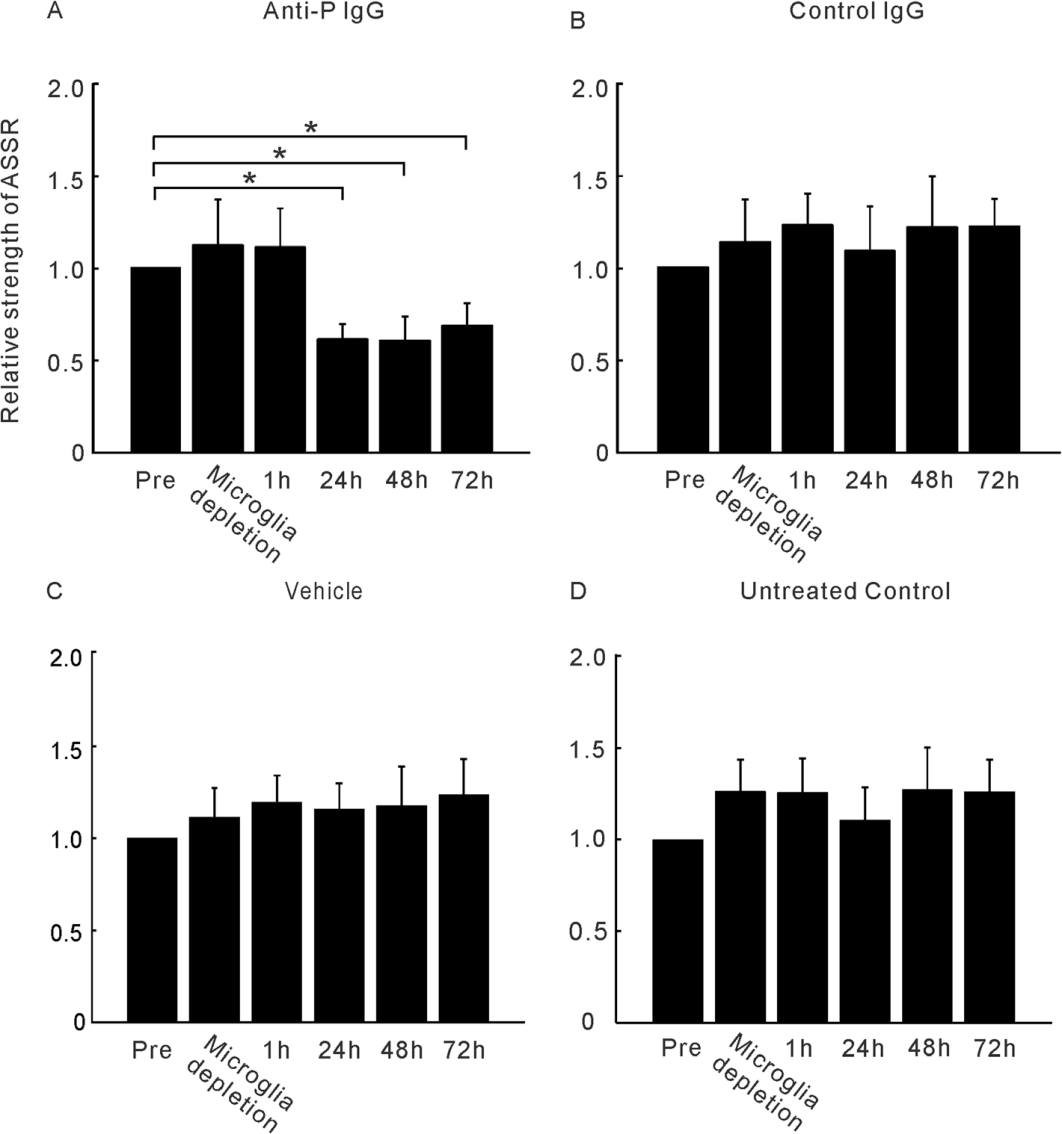
ASSR strength of the microglia-depleted mice at different time points after received the treatment of anti-P (**a**), control IgG (**b**), vehicle (**c**), and untreated control (**d**). The same format as Fig. 2 (*n* = 6)

In this experiment, mice underwent electrode implantation surgery and EEG recording before microglial depletion. After 21 days of microglial depletion, EEG recordings were conducted to evaluate the effect of microglial depletion on the ASSR. The mice then underwent BBB disruption and IgG transfer (*n* = 6 for each group). EEGs were recorded again 1, 24, 48, and 72 h after IgG transfer. No significant changes were observed between the ASSRs recorded before and after microglial depletion (Fig. 6). In contrast, marked and continuous decreases in the strength of ASSRs were observed beginning 24 h after anti-P IgG injection (Fig. 6a). A few unstable changes were observed in the groups treated with control IgG (Fig. 6b) or vehicle (Fig. 6c) and in the untreated controls (Fig. 6d).

### Absence of microglia results in increased neural injury

**Fig. 7:**
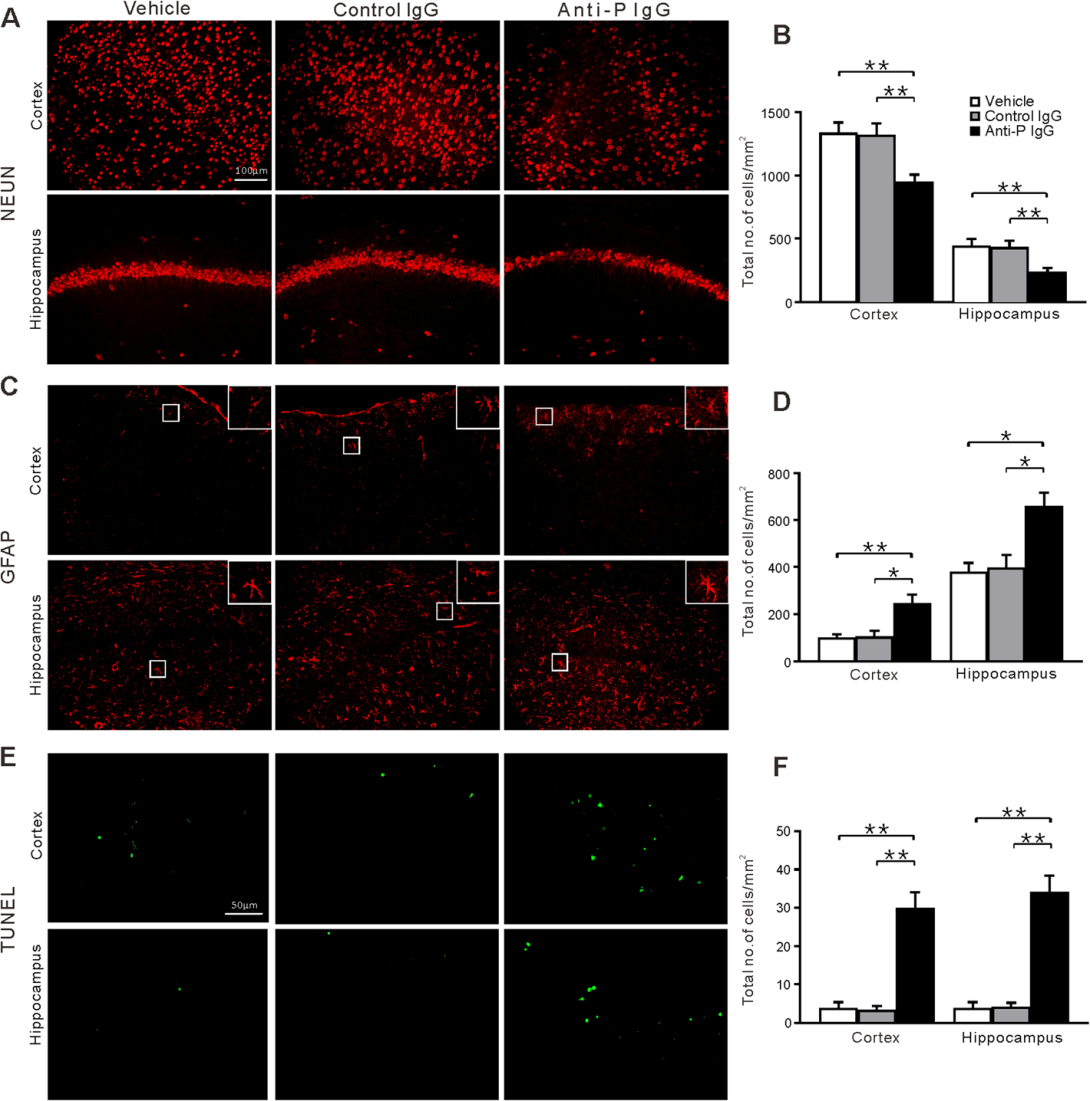
Immunohistochemical analysis of brain section in the microglia-depleted mouse following administration of vehicle, control, or anti-P IgG. The same format as Fig. 3. **a** Representative microphotographs showing NeuN-immunopositive cells. **b** Quantitative analysis of the number of NeuN-immunopositive cells. **c** Representative microphotographs showing GFAP-immunopositive cells. **d** Quantitative analysis of the number of GFAP-immunopositive cells. **e** Representative TUNEL staining. **f** Quantitation of the number of TUNEL positive cells

Immunofluorescence analysis showed that the absence of microglia resulted in a striking decrease in the number of NeuN-immunopositive neurons in the anti-P injection group compared to that in the vehicle and control IgG groups (Fig. 7a and b, *n* = 6 for each group). The density of GFAP-immunopositive astrocytes was also significantly increased, and the astrocytes showed an activated morphology (Fig. 7c and d). The TUNEL assay revealed obvious signs of apoptosis in the hippocampus and cortex (Fig. 7e and f). These results suggest that microglia may protect neurons from the attack of anti-P IgG.

## Discussion

The primary goal of this study was to examine whether circulating anti-P autoantibodies in the blood interfere with neural electrical activity and investigate the defensive mechanisms of the brain. Herein, we provide evidence that circulating anti-P antibodies increase the strength of the ASSR when the BBB is disrupted, allowing access to neurons in the CNS. Although in vitro experiments showed that anti-P antibodies have the potential to induce neuronal apoptosis as a result of calcium overload [16], we found no detectable neuronal apoptosis in the healthy mice that received transfer of anti-P antibodies into the blood circulation. However, the microglia were activated. When microglia were depleted, anti-P IgG caused a serious reduction in the ASSR, obvious neural apoptosis, and astrocyte activation, suggesting a protective effect of microglia on neuronal function. Our results greatly expand the knowledge of the features of the CNS that are related to the presence of anti-P antibodies and highlight the defensive function of microglia in NPSLE.

### Anti-P antibodies and NPSLE

According to previous reports, the prevalence of anti-P antibodies in SLE patients ranges from 6% to 36% [6, 38–40]. A link between anti-P antibodies and CNS dysfunction was first suggested by Bonfa et al. [8], who observed high titers of anti-P antibodies in lupus patients with psychosis. Since this finding, some studies have confirmed an association between anti-P antibodies and the psychiatric manifestations of psychosis and depression [40–46]. In addition, Yoshio et al. found a strong association between anti-P antibodies and other neuropsychiatric presentations (seizures, coma, transverse myelopathy, and aseptic meningitis) [47]*.* The cellular mechanism underlying such anti-P antibody-induced alterations remains a fundamental unresolved question. In vitro studies have suggested that the primary effect of these antibodies is due to direct cytotoxic action on neurons that is initiated by increased calcium influx [15, 16].

One approach to examine in vivo brain activity is EEG. The deleterious effect of antibodies on EEG signals has been shown in previous reports [17, 48–50]. Our present study provides two advantages over previous studies. First, previous studies adopted a method of directly injecting antibodies into the brain parenchyma or ventricle; in contrast, we injected anti-P antibodies intravenously into mice with BBB disruption to assess the pathogenic role of circulating autoantibodies. BBB disruption has been well reported in SLE patients [51], suggesting that circulating anti-P autoantibodies can enter the CNS through the disrupted BBB and interrupt brain function. Our results provide effective evidence for this possibility. Second, previous studies only evaluated alterations in spontaneous EEG activity. In SLE patients, EEG is not a very accurate method of detecting CNS dysfunction [52]. However, abnormalities in event-related potentials have been observed in SLE patients with emotional lability [53]. Therefore, we evaluated brain activity using an evoked EEG signal, the ASSR. Periodic sounds simultaneously evoke a stable oscillatory response called the ASSR at different levels of the auditory system, which phase-locks to the periodic stimulus [18]. The ASSR paradigm is widely used in clinical studies of psychiatric disorders, such as schizophrenia [54] and bipolar disorder [24]. It has been shown that the ASSR is modulated by the state of arousal during anesthesia [55] and emotion state [56]. Here, we recorded the ASSR from awake mice, thus avoiding the effects of anesthesia and emotional state.

The exact mechanism by which anti-P antibodies cause neuronal damage has not been elucidated, but some studies have reported that anti-P antibodies mediates a deleterious effect after binding to a neuronal protein involved in glutamatergic synaptic transmission [16, 57]. In vitro and in vivo studies of the ASSR have also suggested that glutamatergic transmission is critical for neural synchronization [58] and may be involved in the production of the ASSR [59]. Therefore, the abnormalities of the ASSR observed in this study may have been due to glutamatergic transmission dysfunction induced by anti-P antibodies. This possibility is worthy of further investigation.

Another important finding of our study is that microglia play a protective role against anti-P antibody-induced neural injury. Microglia are the resident immune cells of the CNS, representing approximately 5–12% of all CNS cells in the healthy brain [60]. Physiologically, microglia extend long branched processes to sample their microenvironment and monitor the health of surrounding cells. The core function of microglia is the recognition, engulfment, and degradation of extracellular material via phagocytosis [61]. Whether microglial phagocytosis plays a detrimental or protective role in brain diseases remains controversial. On the one hand, efficient clearance of harmful debris is critical for CNS homeostasis. This crucial beneficial role of microglial phagocytosis in axonal regeneration has been shown during brain development [62, 63] and recovery from brain injury [64–66]. On the other hand, inappropriate phagocytosis of synapses in Alzheimer’s disease has been postulated based on mouse models [67, 68].

Here, we identify microglia as important protective components of CNS function. Our results showed that the selective removal of microglia led to marked deterioration of the ASSR after anti-P antibody transfer, which was associated with increased neuronal apoptosis. We used CSF1R blockade to deplete microglia in adult mice. CSF1R is a requisite growth factor receptor for microglia [69], and microglia in the adult brain are fully dependent upon CSF1R signaling for their survival [36]. It has been demonstrated that the systemic application of CSF1R inhibitors leads to the elimination of virtually all microglia from the adult CNS with no ill effects on or deficits in behavior or cognition [36]. Because microglia are the only cell type that expresses CSF1R in the brain [37, 69], the depletion of microglia has minimal effects on other cell types in the CNS and does not lead to inflammation. CSF1R is also expressed by macrophages and osteoclasts [70]. CSF1 regulates the proliferation, differentiation, and survival of macrophages [70], and mice lacking either CSF1 or CSF1R show reduced macrophage density [71]. However, in wild-type mice, PLX3397 treatment only eliminates tumor-associated macrophages and has modest effects on macrophage numbers in other tissues [72]. Thus, the effect of PLX3397 on macrophages is not a main issue in this model.

It is important to note that none of the previous studies combined selective microglial depletion with EEG recording in vivo to link microglial function with neuronal injury. Our results provide convincing evidence supporting a protective effect of microglia on anti-P antibody-induced neural injury. Since microglial phagocytosis is an important defense mechanism in the CNS, we hypothesize that microglia may recognize and engulf the invaded anti-P IgGs to protect neurons. Taken together, these results suggest that the increase in ASSR strength observed in the present study may be due to the hyperfunction of glutamatergic transmission induced by anti-P antibodies. Such a change was temporal and reversible because of the protective function of microglia, which can engulf and remove anti-P IgG. It was confirmed that no obvious neuronal damage was observed in our immunofluorescence study. Once protection by microglia is lost, anti-P IgG can cause irreversible neuronal damage reflected by a decrease in ASSR strength. It is still possible that microglia may play a neuroprotective effect through various other functions, such as direct contact with neurons, the clearance of redundant neurotransmitters, and regulation of the levels of neurotrophic and angiogenic factors [73–76]. These possibilities and the underlying molecular mechanisms need to be clarified in the future. Nevertheless, our present study highlights the protective effect of microglia in anti-P-associated NPSLE and suggests that the ASSR is a convenient and useful method to monitor neural dysfunction.

## Conclusion

We found that circulating anti-P antibodies can act on neurons in the CNS, leading to abnormalities in evoked electrical brain activity, and that microglia protect against this neuronal injury. Therefore, selectively targeting microglia-neuron interactions could have a critical impact on the understanding of the pathophysiology of NPSLE and the development of novel therapeutic approaches.

## Data Availability

The datasets used and/or analyzed during the present study are available from the corresponding author on reasonable request.

## Abbreviations

A1: primary auditory cortex
Anti-P: anti–ribosomal P
ASSR: auditory steady state response
CNS: central nerve system
CSF1R: colony-stimulating factor 1 receptor
EEG: electroencephalogram
NPSLE: neuropsychiatric systemic lupus erythematosus
SLE: systemic lupus erythematosus
